# A tale of two sit-bones: The cyclist’s ischial hygroma (Perineal nodular induration)

**DOI:** 10.17159/2078-516X/2019/v31i1a5641

**Published:** 2019-01-01

**Authors:** J Swart, R V P de Villiers, F Roux, F Rademan, G Thom

**Affiliations:** 1 Division of Exercise Science & Sports Medicine. Department of Human Biology, University of Cape Town, South Africa; 2 Sports Science Radiology, Winelands Radiology, Sports Science Institute of South Africa. Boundary Rd, Newlands, South Africa; 3 Tygerberg Academic Hospital, Cape Town, South Africa; 4 Suite 4, Mediclinic Durbanville, 9 Paul Kruger Road, Durbanville, Cape Town, South Africa; 5 Private Practice, Claremont, Cape Town, South Africa

**Keywords:** cycling, perineal injury

## Abstract

The ischial hygroma, also known as a perineal nodular induration, is a relatively rare and mostly cycling-specific injury that is often incorrectly diagnosed and managed. Here two cases with divergent managements are described to highlight the spectrum of treatment available to manage this condition. The presentation, assessment and management of two cases of perineal nodular induration are discussed.

The management options, namely surgical excision vs conservative management, with saddle pressure mapping highlight that there is no single optimal method and that a multidisciplinary approach should be applied to treat these injuries successfully. Perineal nodular induration should be investigated appropriately to exclude less benign causes of perineal masses. Conservative management and surgical excision can both be successful. Clinicians should be familiar with the assessment and management of this relatively rare but debilitating condition in competitive cyclists.

The biker's nodule or ischial hygroma is a relatively unique pathology that most commonly affects competitive male cyclists and to a lesser extent, females. There are also records of this condition occurring in female equestrians ^[1]^, as well as one interesting but unusual case of it occurring in a lawnmower tester. ^[2]^ These lesions are known by several other terms, including a third or supernumerary testicle, accessory testicle, perineal hygroma or induration. ^[1,3,4]^ Perineal nodular induration was first described by Vuong et al. in 1988 who described two cyclists with this condition. ^[3]^

As previously mentioned, a typical biker's nodule occurs mainly in male competitive cyclists. Symptoms include pain on pressure, such as when sitting in the saddle. A dull constant ache even when not riding has also been described. ^[1]^ In one of this study’s cases, the patient also had symptoms due to the close proximity to the sciatic nerve. Typically, a nodule is palpable on one or both sides of the midline, posterior to the scrotal sac, usually 2–3 cm in size. ^[2, 4, 5]^ This nodule is typically fibroelastic and may adhere to the adjacent connective tissue. ^[6]^ In females, the nodules are usually unilateral and may occur in the typical location as described above or may occur in either or both of the labium majora.^[7]^

The lesion is believed to be caused by increased friction and vibration between the ischial tuberosities and the hard saddle as a result of the constant rubbing of the superficial perineal fascia against bony structures.^[2,7,8]^ Thus mountain bikers are regarded as a particularly high-risk group for developing these lesions. ^[9]^

The variant which occurs in the labia majorus may show an admixture of adipose tissue, variably cellular hyalinised tissue containing bland, spindle-shaped fibroblasts, blood vessels, and nerve fibres. In some areas, thick cords of fibrous tissue imparting a keloid-like appearance can occur. Other histologic features include plump mesenchymal cells with round or ovoid nuclei and abundant eosinophilic cytoplasm resulting in an epithelioid, plasmacytoid, or ganglion-like appearance, lymphocytic infiltrate around blood vessels, foci of fat necrosis, and collections of elastic fibres ^[7]^.

Two typical cases of perineal nodular infiltration are described which are managed with differing modalities.

## Case report 1

### History

A 45-year-old competitive female cyclist presented to her sports physician with a year-long history of a dull ache in the left inner buttock which radiated to the left hamstring. The pain was aggravated by cycling and sitting on her chair at work. She had initially been treated unsuccessfully by a physiotherapist for hamstring tendinosis.

### Examination

No visible abnormality was detected. On palpation, a nodule of approximately 2 × 2 cm was noted adjacent to the left ischial tuberosity. It was mildly tender on palpation and not fixed to the underlying muscle or bone.

### Special Investigations

The patient was initially sent for diagnostic high-resolution ultrasound. This was followed by a MRI study of the pelvis.

**Fig. 1:**
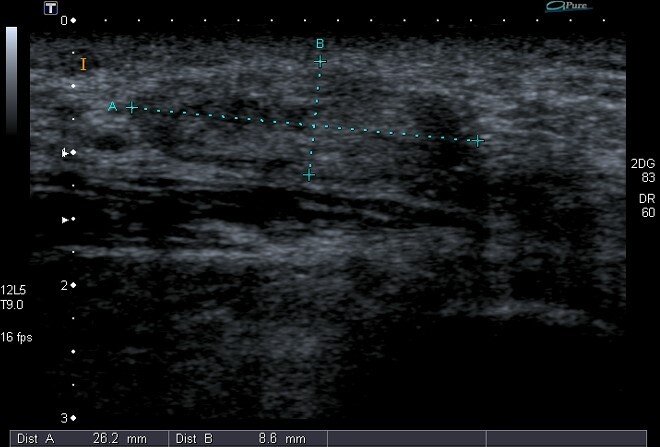
Longitudinal high-resolution greyscale ultrasound image of the perineal region just medial to the ischial tuberosity

**Fig. 2:**
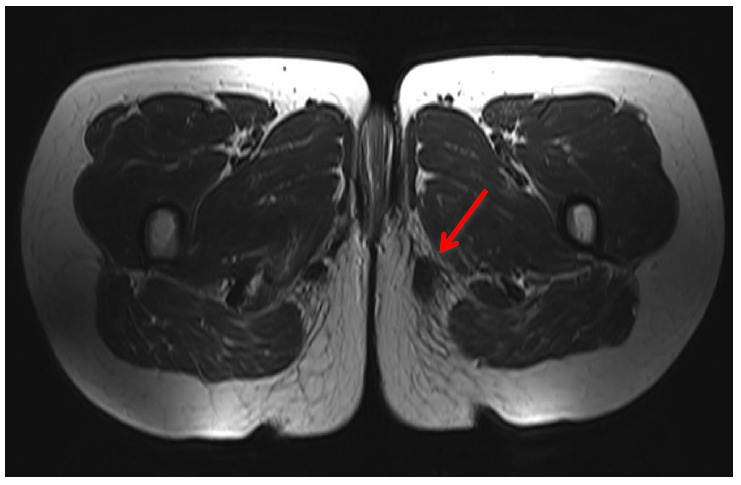
MRI pelvis **-** Axial T1 weighted image of the perineal region, demonstrating a low signal intensity lesion to the left of the midline in the superficial soft tissue of the perineum

High-resolution greyscale ultrasound demonstrated a well-circumscribed predominantly hypo-echoic 2 × 1 cm nodule in the subcutaneous tissue just medial to the ischial tuberosity (Fig. 1). Magnetic resonance imaging showed a fairly well-circumscribed 20 mm × 15 mm mass in the subcutaneous tissues of the left buttock in close relation to the ischial tuberosity and proximal insertion of the hamstring. The lesion was hypointense on all sequences (Fig. 2).

Based on these and clinical findings the diagnosis of an ischial hygroma was made.

### Management

The sports physician injected a combination of local anaesthetic and corticosteroid into the nodule. There was no subsequent improvement. She was then referred for surgical excision of the lesion.

Dissection through the skin and subcutaneous tissue revealed a fibrotic mass approximately 3 cm in size situated medial to the sciatic nerve and superficial to the hamstring origin at the ischial tuberosity. A white focus was noted centrally within the lesion compatible with the injected corticosteroid.

**Fig. 3:**
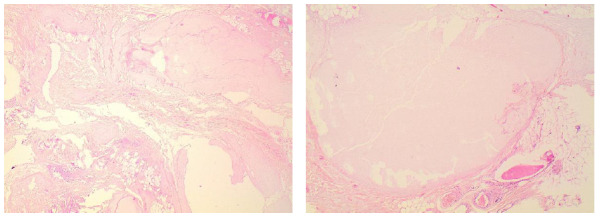
Histological characteristics of perineal nodular induration demonstrating dense hyaline tissue and pseudocystic changes

Histology revealed a combination of dense hyalinised fibrous tissue, surrounding oedematous fibrous tissue and pseudocystic area compatible with a cyclist nodule (Fig. 3). The patient initially had an uneventful recovery and was asymptomatic. However, at a two year follow-up she had not returned to competitive cycling due to persistent discomfort, albeit less than at the time of presentation.

## Case report 2

### History

A 61-year-old competitive male cyclist presented to these authors’ clinic with a history of insidious onset pain in the left perineal area which occurred during cycling. Shortly prior to seeking medical attention, he noted a tender mass in his perineum.

### Examination

No visible abnormality was detected. On palpation, a nodule of approximately 1 cm in transverse and 4 cm in longitude was noted adjacent to the left ischial tuberosity. It was firm and rubbery in consistency, mildly tender on palpation, and not fixed to the underlying muscle or bone.

### Special Investigations

The patient was referred for diagnostic high-resolution ultrasound.

High-resolution greyscale ultrasound demonstrated a well-defined, heterogeneous, predominately cystic mass inferior to the left scrotum. It measured 5.0 × 2.4 × 4.0 cm and contained hypoechogenic material within it, with no increased vascularity.

Based on the imaging and clinical findings, it was diagnosed as an ischial hygroma.

### Management

The patient was reluctant to undergo surgery and specifically requested conservative management.

A comprehensive assessment of his cycling biomechanics and saddle contact point was performed.

Pre-fitting and static kinematics were performed using ErgoFiT^TM^ (http://www.sciencetosport.com/ergofit) software and kinematic tools. Saddle pressure mapping was performed using Gebiomized**^®^** dynamic saddle pressure mapping systems (http://www.gebiomized.de).

Salient findings were that underwear was being worn under the cycling-specific garment and that the cycling-specific garments were of relatively low quality, with chronic wear demonstrated on the padding material. The kinematic assessment demonstrated excessive saddle height (static knee flexion angle (KFA) of 22**°** using the Holmes^10^ method) and excessive saddle setback (10.5 cm). This was confirmed on initial saddle pressure mapping by a front: rear pressure balance ratio of 62:38.

**Fig. 4:**
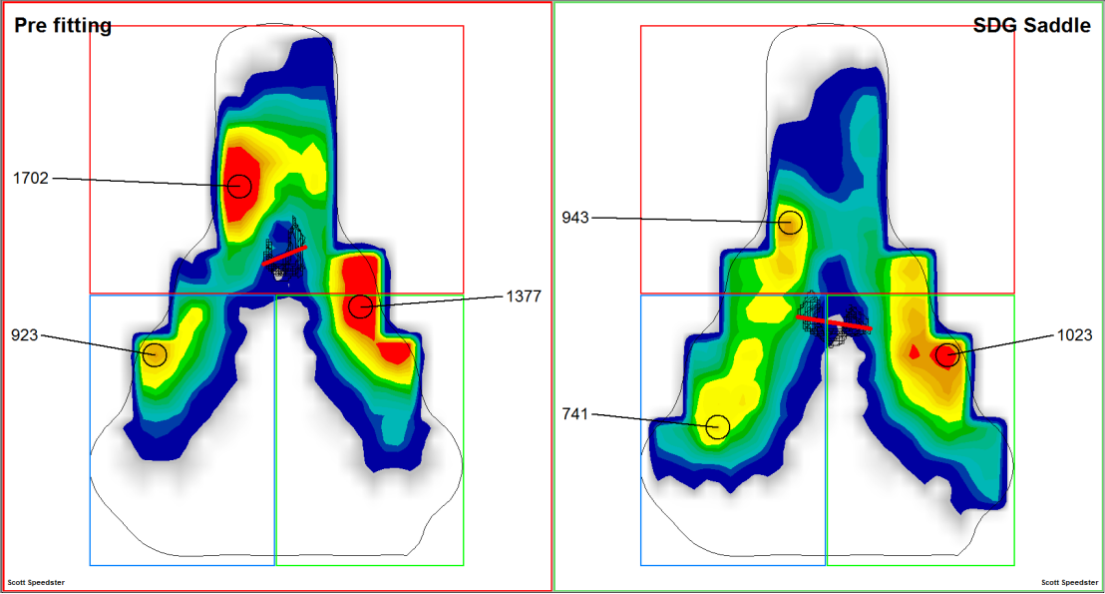
Pre- and post-intervention saddle pressure mapping demonstrating increased contact area, reduced peak pressures and more favourable pressure distribution

After correction of the kinematics (KFA of 31**°** and saddle setback of 6.8 cm) a series of minor adjustments were made and numerous different saddle types were tested to achieve the most favourable saddle pressure mapping possible (pre- and post-pressure mapping are shown in Fig.4).

Pre- and post-changes (Table 1) improved overall pressure distribution by:

- Improving front: rear pressure balance ratio from 62:38 to 46:54
- Increasing loaded area in left ischial area from 4300 mm^2^ to 5325 mm^2^
- Reducing peak pressure from 923 mbar to 724 mbar

The patient returned to cycling activity and has subsequently not experienced any recurrence of symptoms at two-year follow-up.

## Discussion

These two cases illustrate several important points.

Firstly, the nodule may not be initially readily apparent and the symptoms in Case 1 were originally thought to be related to the tendinous insertion of the hamstring and sciatic nerve, which lie in close proximity. It is therefore important for clinicians to be aware of this unique entity and to have a high index of suspicion which can improve early management and prevent unnecessary medical treatment, months of frustration and potential loss of income.

Imaging characteristics are rarely reported; however, an example can be found in a 2009 article by Van de Perre et al.^[4]^ Ultrasound demonstrates a fairly well-circumscribed hypoechoic nodule with an absence of the Doppler signal. Small internal cystic areas may also be seen. CT (computed tomography) and MRI (magnetic resonance imaging) show lack of contrast uptake, which explains the hypovascular nature as seen on histology examinations.^[4,7]^ On an MRI, the lesion would be hypointense on all sequences. The primary role of imaging is to determine the location and exact extent of the lesion and to exclude rarer and other important conditions, such as aggressive angiomyxoma.^[6]^ In most clinical scenarios, ultrasound would suffice. ^[4]^

If the diagnosis is unclear, imaging plays an important role in the differentiation from other causes of perineal swelling. These would include an abscess, epidermal cyst, cutaneous adnexal mass, lipoma or tumour (sarcoma or metastases).^[2, 4, 8]^ Non-mass enhancement on MRI is a reassuring feature and readily excludes malignancy.^[4]^ Vuong et al. states that this pseudoneoplastic lesion has a relatively constant appearance, but if not realised to be a benign nonneoplastic lesion, there is a potential for misdiagnosis. ^[3]^ This again stresses the importance of suspecting this lesion in the correct clinical context and using imaging as an important diagnostic tool.

Secondly, the management of this entity can be successful by using both conservative and more invasive methods.

In these authors’ experience, most clinicians limit management to avoidance of the causative factor, be it cycling or horse riding. This is sometimes not a practical option and, in these authors’ limited experience, it is generally not sufficient as therapy. ^[4]^ Correct bike setup, saddle type and saddle position will go a long way in relieving symptoms and preventing recurrence of the lesions. Special attention should also be given to cycling shorts and the construction of the padding.^[4]^ Local infiltration with corticosteroids or hyaluronidase are described as first-line invasive treatment. If none of these therapies are efficacious, surgery should be considered. ^[3–5, 8]^

Conservative management includes a period of rest from cycling to allow the area to settle which should reduce the size of the lesion but not eliminate it. Once the lesion has settled a comprehensive assessment of kinematics and saddle contact point can reduce loading of the area and prevent recurrent episodes of pain and enlargement. However, this requires dedicated expertise in the field of cycling biomechanics and specialised equipment to assess the saddle/cyclist interface. Clinicians should enquire about such expertise in their area and refer to the appropriate experts for management.

In these authors’ experience, an injection of the lesion with corticosteroids has not resulted in lasting relief but can provide a temporary treatment option or adjunct to more specific management of the kinematics and contact area analysis. Clinicians should be aware of the risk of fat necrosis at the injection site which may cause further discomfort on the commencement of cycling.

Failure to resolve symptoms using conservative measures or a lack of expertise in this field in the clinician’s geographical area should warrant a referral for surgical excision. Surgical excision of large hygromas can be technically challenging due to the poorly defined boundary of the lesion and adherence to surrounding structures. As such, the patient may often experience a residual nodule, albeit smaller in size, and experience some persistent discomfort in the area post-surgery. The histopathological features show a myxoid degeneration of the fatty tissue, often with a pseudocyst formation. This is surrounded by dense hyalinised fibrous tissue which merges towards the periphery with oedematous fibrous tissue containing fibroblast-like spindle cells, thick collagen bundles, degenerate elastic fibres, and clusters of capillaries which may show diapedesis of erythrocytes or even haemorrhagic foci. ^[2,4]^ However, the lesion is not well vascularised. ^[4]^ The margins of the lesion are not well circumscribed and there is no synovial lining. ^[2,6,11]^ Focally, spindle cells showed mild degenerative atypia reminiscent of that seen in ischaemic fasciitis.

In summary, clinicians should have a high index of suspicion for perineal nodular induration in cyclists presenting with perineal pain or pain in adjacent areas. They should be aware of the potential for more serious pathology and use imaging to differentiate these less benign lesions. Lastly, management principles can be dictated by the availability of expertise in cycling kinematics in their area but multiple management approaches can result in successful outcomes for the patient.

## Figures and Tables

**Table 1:** Saddle pressure mapping values pre-/post-optimisation

Parameters (pre-/post)	Pubic Bone	Left ischium	Right ischium
Max. pressure [mbar]	1702/943	923/741	1377/1023
Mean pressure [mbar]	400/267	210/313	281/304
Loaded area [mm2]	6400/6200	4300/5325	4475/5775

Pressure front/rear		62:38/46:54	
Pressure left/right		43:57/51:49	
